# Correlation analysis of systemic immune inflammation index with the occurrence and clinical outcomes of hypertension: a systematic review and meta-analysis

**DOI:** 10.3389/fcvm.2025.1593481

**Published:** 2025-11-10

**Authors:** Shude Sun, Jiamei Fu, Jianfei Yang, Liang Zhao, Boyan Zhao, Yabin Zhou

**Affiliations:** 1 School of Graduate Students, Heilongjiang University of Chinese Medicine, Harbin, Heilongjiang, China; 2 The Second Department of Cardiovascular Diseases, First Affiliated Hospital, Heilongjiang University of Chinese Medicine, Harbin, Heilongjiang, China

**Keywords:** NEUT count * PLT count/LYM count, hypertension, the occurrence of hypertension, prognosis for patients with hypertension, meta-analysis

## Abstract

**Objective:**

This systematic review and meta-analysis aimed to examine the link between the systemic immune inflammation index (SII) and the incidence and clinical outcomes of hypertension (HTN).

**Methods:**

Studies on the link association SII levels with the incidence and prognosis of HTN were retrieved in PubMed, Embase, Web of Science, and Cochrane Library databases. The standardized mean difference (SMD) was employed to discuss the stability of the results and potential sources of heterogeneity. The meta-analysis was performed with Review Manager 5.4.1 and STATA 15.0 software.

**Results:**

In total, 19 articles were included, covering 187,195 patients. The results demonstrated that elevated SII was associated with the incidence of HTN (continuous variable: SMD = 1.22, 95% confidence interval [CI]: 0.56, 1.89, *P* = 0.000; categorical variable: odds ratio [OR] = 1.14, 95% CI: 1.08, 1.20, *P* = 0.000). Furthermore, SII was also closely linked to the prognosis of HTN patients. Subgroup analyses based on study design, sample size, region, and mean age revealed that high SII levels were associated with the incidence and prognosis of HTN. Compared to the low SII group, the incidence of HTN was greater in individuals with high SII (continuous: SMD = 1.22, 95% CI: 0.56, 1.89, *P* = 0.000; categorical: OR = 1.14, 95% CI: 1.08, 1.20, *P* = 0.000). HTN patients in the high SII group had higher rates of mortality, major cardiovascular adverse events, carotid intima-media thickness, and asymptomatic organ damage than those in the low SII group.

**Conclusion:**

SII is potentially associated with the risk and prognosis of HTN, and is likely to become a valuable inflammatory marker for preventing HTN. In light of the inherent limitations of this study, more prospective, large-scale studies are necessary to confirm the findings of this study.

**Systematic Review Registration:**

https://www.crd.york.ac.uk/PROSPERO/, PROSPERO CRD42024618091.

## 1 Introduction

According to the Global Burden of Disease Study, it is crucial to manage abnormal blood pressure, which is a major risk factor for mortality worldwide (1). Hypertension (HTN) and related complications, like ischemic heart disease and stroke, rank as the first and second leading Level 3 causes of death worldwide, contributing to 16.2% and 11.6% of deaths, respectively. In 2019, stroke caused around 6.55 million deaths (2). HTN is the most prevalent cardiovascular disease (CVD) and a high-risk factor for many other CVDs. Long-term inadequate control of blood pressure can eventually result in heart, brain, and kidney complications. The pathogenesis of HTN is currently not well understood. Existing studies mainly focus on genetic factors, aortic stiffening (3), the renin-angiotensin-aldosterone system (RAAS), the sympathetic nervous system, vascular remodeling, and other contributing factors. Recently, the association of HTN with inflammation and immune response has become increasingly notable. Evidence indicates that inflammation-linked factors, cells, and markers are associated with the occurrence, progression, and prognosis of HTN (4–6).

Typical risk factors for HTN include age, sex, body mass index (BMI), disease duration, and diabetes. However, these risk factors are tied to several common chronic diseases and may not be specific to the occurrence of HTN. Furthermore, it remains unclear whether these factors impact the prognosis of HTN patients. In addition to the recognized risk factors, systemic inflammation is considered to be a critical factor in the prognosis of various CVDs. Systemic immune-inflammation index (SII) is a novel inflammatory biomarker derived from neutrophil (NEUT), platelet (PLT), and lymphocyte (LYM) counts. It is calculated by the following formula: NEUT count * PLT count/LYM count. The SII incorporates PLT, NEUT, and LYM counts, which help to better illustrate the balance between immune response and inflammation. The prognostic value and feasibility of SII have been proven in various types of cancer, like aortic dissection, peripartum cardiomyopathy, and coronary artery disease (7–12). In a study by Yumeng Shi and Wei Zhou (13) in 2023, spanning from 2017 to 2020 and covering 13,742 participants, it is found that SII can predict the occurrence of HTN and serve as an independent predictor for the onset of HTN. In another study by Ying Chen et al. (14) in 2024, spanning from 1999 to 2018 and covering 44,070 adults, when Ln(SII) is lower than 5.89 (1,000 cells/*μ*l), it is linked to a decreased risk of HTN. However, once it surpasses this threshold, Ln(SII) is tied to an increased risk of HTN.

Currently, a growing body of clinical studies is focusing on the link between SII and the occurrence and prognosis of HTN. Nonetheless, due to variations in sample sizes, populations, follow-up times, and HTN incidence, there is currently no meta-analysis that summarizes the existing clinical data, and no definitive conclusion has been made regarding the link between SII and the occurrence of HTN. Hence, this study aims to conduct a systematic review and meta-analysis to include clinical studies examining the link between SII and the incidence and clinical outcomes of HTN. By merging data from these studies, we seek to provide evidence-based conclusions regarding whether SII can predict the occurrence and prognosis of HTN, ultimately offering theoretical support for preventing HTN and improving its prognosis in clinical practice.

## 2 Materials and methods

### 2.1 Literature search

**Figure 1:**
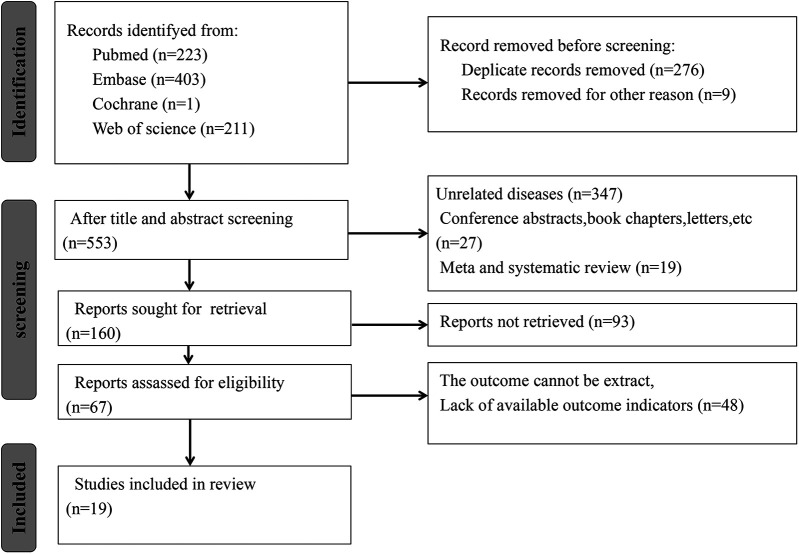
Literature screening process. According to the inclusion criteria, 838 articles were included, and according to the exclusion criteria, 821 articles were excluded. Finally, this meta-analysis included a total of 17 studies.

This study adhered to the guidelines outlined in the Preferred Reporting Items for Systematic Reviews and Meta-Analyses 2020 statement (15), and the research protocol was registered with the International Prospective Register of Systematic Reviews (PROSPERO: CRD42024618091). Two investigators (SSD and FJM) were responsible for developing the search strategy. The subject terms and keywords for searching multiple databases, including PubMed, Embase, Web of Science, and Cochrane Library, were developed. The retrieval time was up to August 15, 2024. Search terms were designed using a wide range of terms, like “systemic immune inflammation index”, “SII”, “Hypertension”, “Blood Pressure, High”, “Blood Pressures, High”, “High Blood Pressure”, and “High Blood Pressures”. Figure 1 presents the literature retrieval strategy.

### 2.2 Study selection

I. Inclusion criteria

(i) Study type: Cohort and case-control studies; (ii) Study subjects: People at risk for or diagnosed with HTN, regardless of sex, age, race, occupation, or ethnicity; (iii) Exposure factor: SII; (iv) Outcome measures: incidence of HTN and prognosis of patients with HTN (at least one of the following clinical outcomes assessed in the acute phase or during follow-up: death, major cardiovascular adverse events [MACE], and carotid intima-media thickness [CIMT]).

II. Exclusion criteria
(i) Non-English studies; (ii) Studies without accessible full text; (iii) Conference papers, newsletters, reviews, and other articles; (iv) Studies with multiple publications or identical data (in this situation, a study with higher methodological quality and more complete data was chosen).

Study selection was independently executed by two investigators (SSD and FJM). Disagreements were resolved through negotiation, and if no consensus was achieved, the issue was addressed by a third investigator (YJF).

### 2.3 Data extraction

Two investigators (SSD and FJM) independently extracted data from the included articles, including study information (study title, first author, and publication year), population characteristics (number of study subjects, mean age, BMI, systolic blood pressure [SBP], diastolic blood pressure [DBP], cut-off value of SII, duration of follow-up, and geographic area) and clinical outcomes (death, MACE, CIMT, and others). Disagreements were resolved through negotiation, and if no consensus was achieved, the issue was addressed by a third investigator (YJF).

### 2.4 Risk of bias assessment

The risk of bias in the included studies was independently appraised by two investigators (SSD and FJM) using the Newcastle-Ottawa Scale (NOS) (16), and the results were cross-checked. Disagreements were resolved through negotiation, and if no consensus was achieved, the issue was addressed by a third investigator (YJF).

### 2.5 Statistical analysis

Categorical variables were expressed as odds ratios (OR) and 95% confidence intervals (CI), and continuous variables were expressed as standardized mean differences (SMD) and 95% CI. The meta-analysis was performed using Review Manager 5.4.1 and STATA 15.0 software. Heterogeneity among the included articles was appraised utilizing the Cochrane Q test and I^2^ test, with a significance level set at *α* = 0.05. *P* ≤ 0.1 or I^2^ ≥ 50% suggested a high degree of heterogeneity among the studies. All meta-analyses were executed using a random-effects model, and subgroup analyses were performed to examine potential sources of heterogeneity. Additionally, a sensitivity analysis was carried out utilizing the leave-one-out method to appraise the impact of each study on the pooled effect size. Egger's test was used to assess publication bias. *P* < 0.05 indicated publication bias, and a corresponding funnel plot was generated.

## 3 Results

### 3.1 Literature screening process and results

A total of 838 potentially relevant studies were identified from the four databases. Duplicates and ineligible articles were deleted, and 553 articles were left. Upon reading the titles and abstracts, 393 studies were excluded based on predefined inclusion and exclusion criteria, including 347 unrelated studies, 27 conference abstracts, book chapters, and letters, and 19 meta-analyses or reviews. The full texts of the remaining 160 studies were retrieved. However, the full texts of 93 studies were not retrieved, and 48 studies had no usable data. Ultimately, this meta-analysis included 19 articles (13, 14, 17–31) (Figure 1).

### 3.2 Basic characteristics of the included studies

The minimum sample size was 91, while the maximum sample size was 64,500. (Supplementary Table S1) Out of the 17 studies, 9 were prospective and 8 were retrospective. Complications encompassed diabetes mellitus (DM), dyslipidemia, cerebral infarction, carotid intima-media thickness (CIMT), coronary heart disease (CHD), cancer, heart failure, and chronic obstructive pulmonary disease (COPD). Among these complications, DM and cerebral infarction were primary. Of the included studies, one used death as the endpoint, one used CIMT, one focused on asymptomatic organ damage, and one reported MACE. The SII value was divided into two groups in 9 studies, three groups in 5 studies, and four groups in 3 studies. Every study had an NOS score of 6 or higher.

### 3.3 Meta-analysis results

#### 3.3.1 Performance of SII for predicting HTN

##### 3.3.1.1 Continuous variable

**Figure 2:**
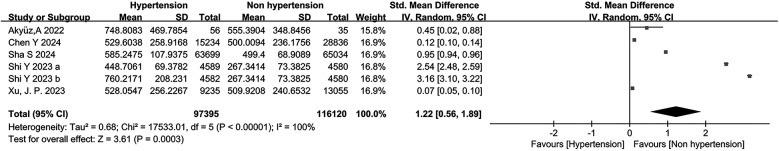
Forest plot for continuous variable. The heterogeneity test results of continuous variables showed significant heterogeneity among the included studies (I^2^ = 100%, *P* = 0.0003). The SII level in patients with hypertension was significantly higher than that in non-hypertensive individuals (SMD = 1.22, 95%CI: 0.56, 1.89, *P* = 0.000).

Six studies consecutively evaluated the link between the development of HTN and the levels of SII as a continuous variable (116,120 non-HTN patients and 97,653 HTN patients). Large heterogeneity was noted between the studies (I^2^ = 100%, *P* = 0.0003) (Figure 2). Consequently, a random-effects model was utilized for the meta-analysis. The meta-analysis revealed that the SII levels were significantly higher in HTN patients than in the non-HTN population (SMD = 1.22, 95% CI: 0.56, 1.89, *P* = 0.000) (Figure 2).

##### 3.3.1.2 Categorical variables

**Figure 3:**
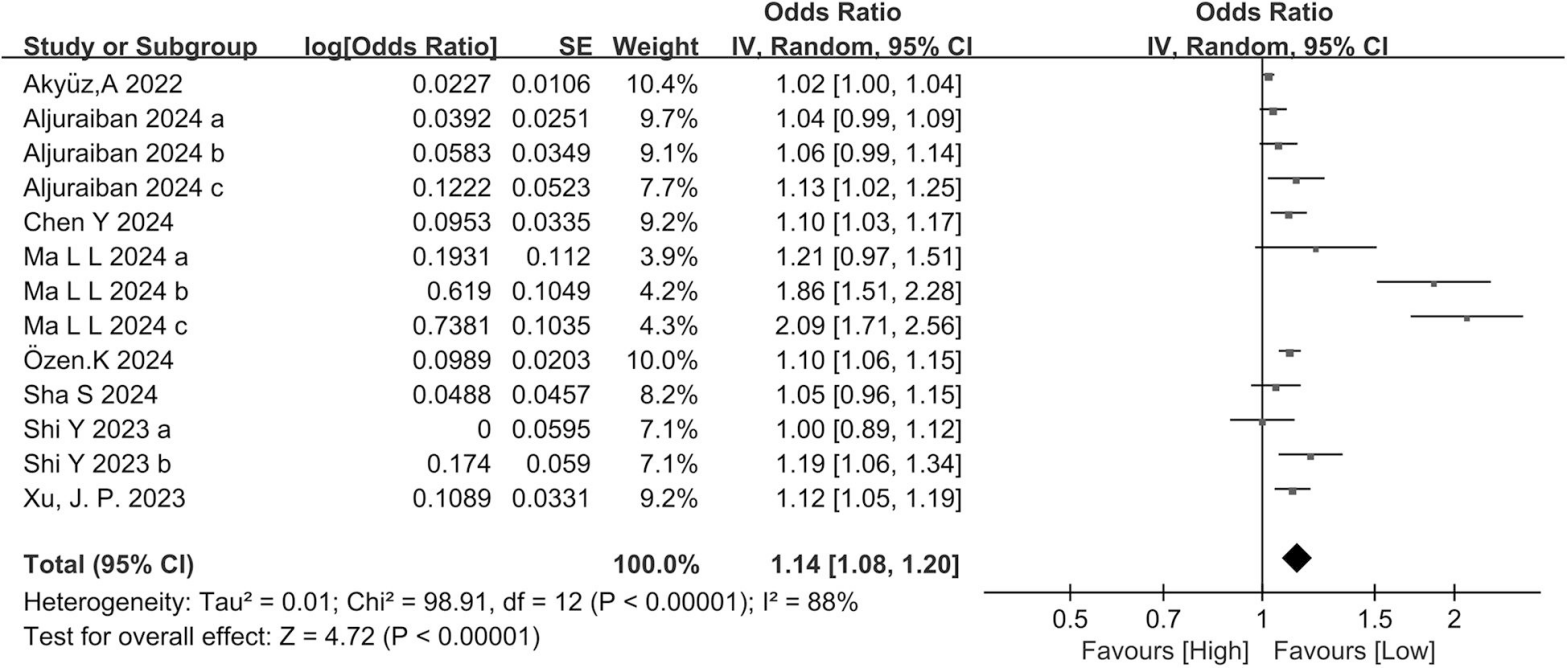
Forest plot for categorical variable. The heterogeneity test results showed significant heterogeneity among the included studies (I^2^ = 88%, *P* < 0.00001). The meta-analysis results showed that patients with high SII values had a significantly increased risk of developing hypertension (OR =  1.14, 95% CI: 1.08, 1.203, *P* = 0.000).

**Figure 4:**
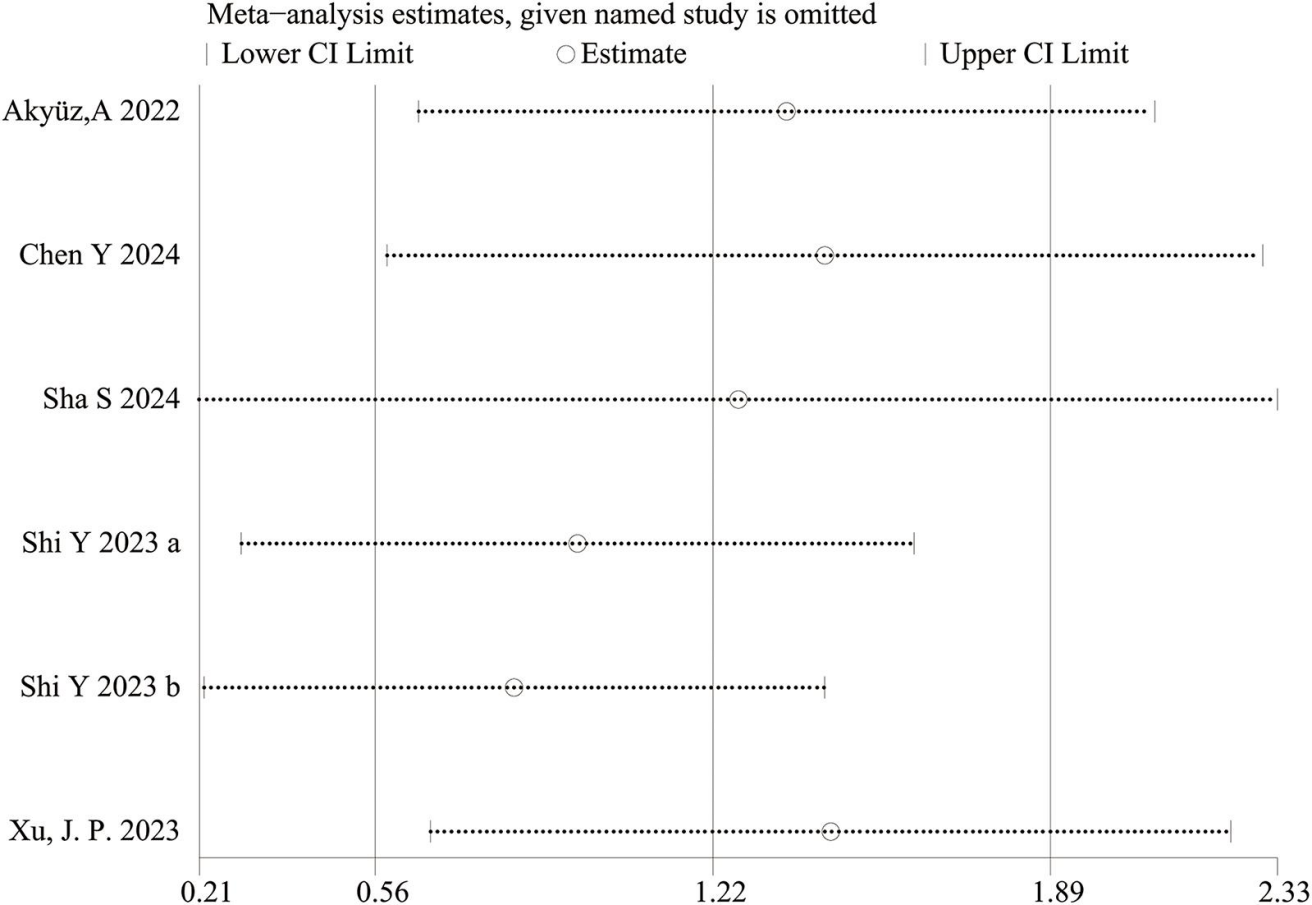
Sensitivity analysis for categorical variable. The sensitivity analysis revealed that the meta-analysis results of SII (categorical variable) as a potential correlated factor for hypertension remained stable (Figure 7), and were not significantly affected by any single study.

Eight studies categorically assessed the association between the occurrence of HTN and SII as a categorical variable in people at risk of HTN. Large heterogeneity was observed among the included studies (I^2^ = 88%, *P* < 0.00001) (Figure 3). Meta-analysis was therefore performed using the random-effects model. The meta-analysis indicated that patients with high SII values had a markedly increased risk of HTN (OR = 1.14, 95% CI: 1.08, 1.203, *P* = 0.000) (Figure 4).

#### 3.3.2 Sensitivity analysis and publication bias

**Figure 5:**
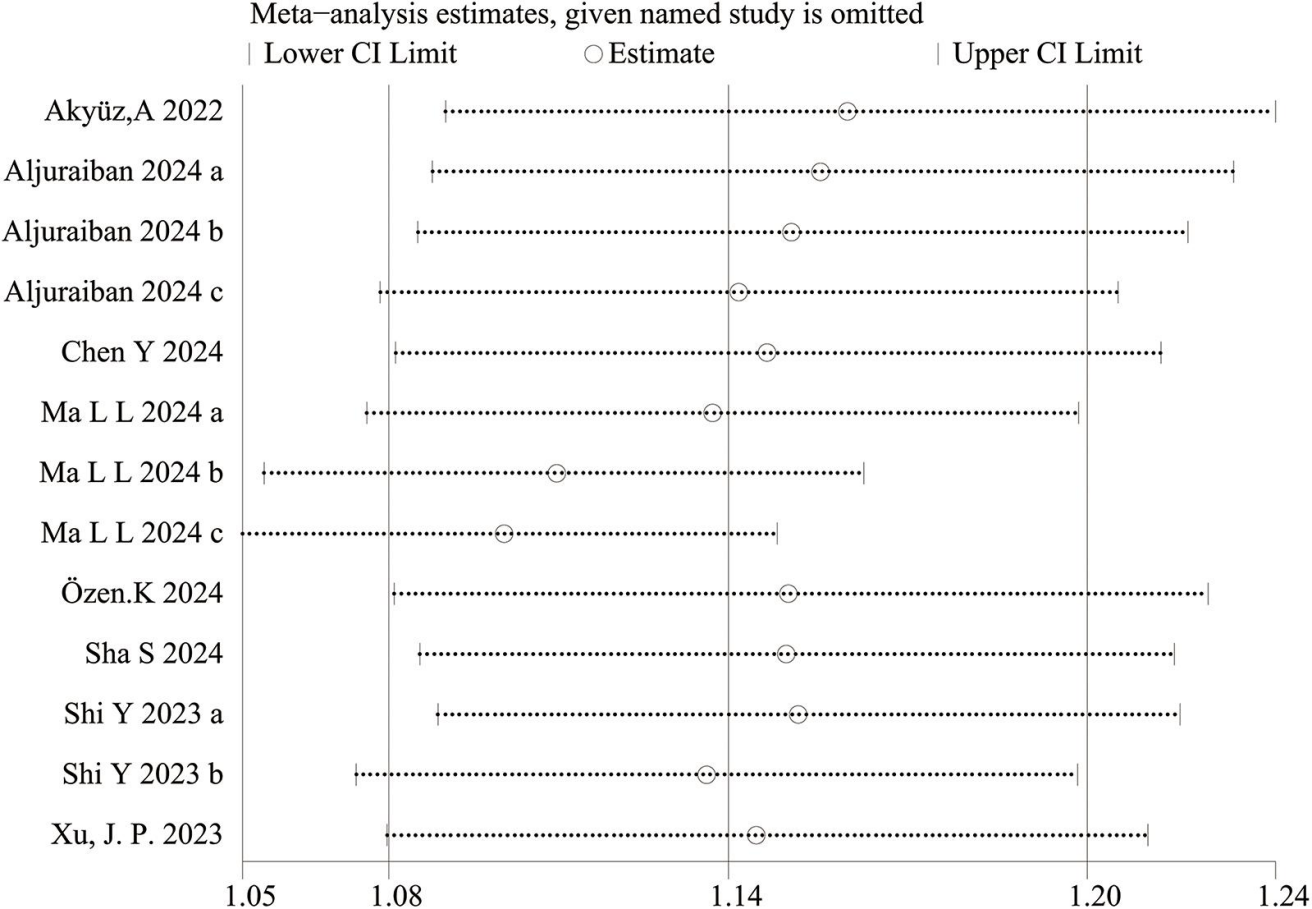
Sensitivity analysis for continuous variable. The sensitivity analysis revealed that the meta-analysis results of SII, as a potential continuous variable associated with hypertension (Figure 6), remained stable and were not significantly influenced by any single study.

**Figure 6:**
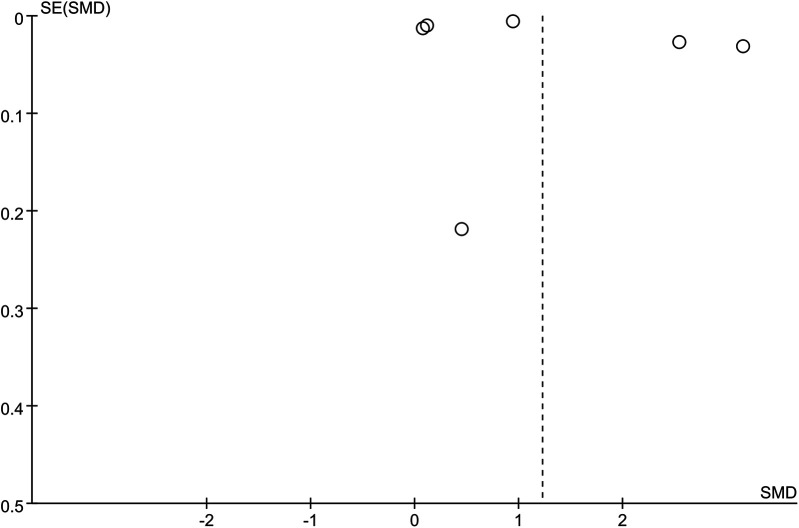
Funnel plot for continuous variable. The funnel plot suggests that SII as a continuous variable for predicting hypertension is symmetrical, indicating no significant publication bias.

**Figure 7:**
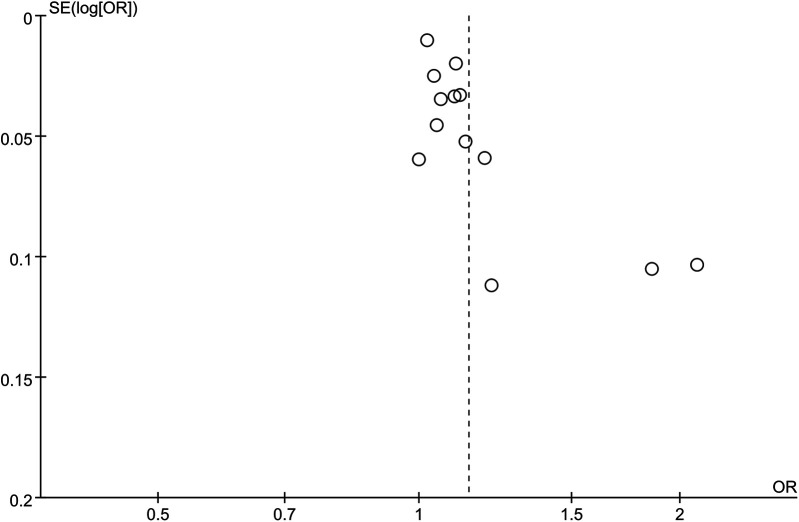
Funnel plot for categorical variable. The funnel plot of SII (categorical variable), as a potential associated factor for hypertension, is asymmetric, suggesting significant publication bias.

The sensitivity analysis revealed that the performance of SII in predicting HTN remained stable, both as a continuous variable (Figure 5) and as a categorical variable (Figure 4). The meta-analysis results were not noticeably influenced by any single study. The funnel plot for studies on SII as a continuous variable for predicting HTN was symmetrical (Figure 6). Nonetheless, for studies with SII as a categorical variable in predicting HTN, the funnel plot was asymmetrical (Figure 7). Moreover, Egger's test indicated that there was no significant publication bias in studies utilizing SII as a continuous variable in predicting HTN (*P* = 0.596). However, when SII was treated as a categorical variable, significant publication bias was observed (*P* = 0.005).

#### 3.3.3 Subgroup analysis

A subgroup analysis for studies utilizing SII as a categorical variable in predicting HTN, was conducted based on study design, sample size, geographical region, and mean age. The results indicated that the predictive performance of SII remained consistent and stable across all subgroups. Furthermore, subgroup analysis indicated that the I^2^ value decreased to 40% within the American subgroup. The detailed results of the subgroup analysis are available in Table 1. In the subgroup analysis by cut-off values of SII as a categorical variable, in the ≥500 group, OR was 1.05 (95% CI: 1.01–1.09; *p* = 0.39\0.01; I^2^ = 0%), while in the <500 group, OR was 1.07 (95% CI: 1.00–1.16; *p* = 0.02\0.07; I^2^ = 69%). According to subgroup analysis by cut-off values of SII as a continuous variable, in the ≥500 subgroup, OR was 2.13 (95% CI: 1.51–2.75; *p* < 0.00001; I^2^ = 99%).

**Table 1:** Subgroup analysis.

Subgroup	Incidence of hypertension (classification)			
	Study	OR [95%CI]	*P* value	*I* ^2^
Total	15	1.02 [1.01, 1.03]	<0.00001	91%
Study design				
Cohort	11	1.18 [1.09, 1.27]	<0.00001	87%
Case-control	4	1.00 [1.00, 1.01]	<0.00001	91%
Sample-size				
≥1,000	9	1.20 [1.10, 1.31]	<0.00001	89%
<1,000	6	1.00 [1.00, 1.01]	<0.00001	88%
Region				
Asia	8	1.20 [1.10, 1.31]	<0.00001	92%
Europe	/	/	/	/
America	4	1.08 [1.02, 1.15]	0.17	40%
Mena/median age				
≥50	3	1.09 [1.01, 1.17]	0.0003	88%
<50	9	1.20 [1.09, 1.31]	<0.00001	89%
SII cut off (classification)				
≥500	3	1.05[1.01, 1.09]	0.39\0.01	0%
<500	3	1.07[1.00, 1.16]	0.02\0.07	69%
SII cut off (continuous)				
≥500	3	2.13[1.51, 2.75]	<0.00001	99%

### 3.4 Qualitative description of SII for predicting the prognosis of HTN patients

In the two case-control studies conducted by Çırakoğlu ÖF and Yılmaz AS in 2021 and Şaylık, F et al. (20, 28) in 2023, the association between SII and the incidence rate of CIMT was evaluated in HTN patients (including 130 HTN patients with CIMT and 301 HTN patients without CIMT). Due to the insufficient number of related studies, a meta-analysis was not conducted. In the two studies, the median and highest values of SII were significantly higher in patients with CIMT than in those without CIMT. This indicated that higher SII was associated with a greater risk of concurrent CIMT, acting as a potential associated factor.

A case-control study published by Cao Y et al. (19) in 2023, spanning from 2011 to 2019 and including 8,524 HTN patients, assessed the association between SII and mortality in HTN patients. Due to insufficient relevant studies, a meta-analysis was not conducted. In their study, SII was divided into quartiles, and the results indicated that both the OR value and 95% CI increased with higher SII values, with all OR values exceeding 1. This result indicated that higher SII was associated with an elevated mortality rate, serving as a potential associated factor for mortality.

Inanc, I., and C. Sabanoglu (21) conducted a case-control study between 2022 and 2021, including 250 patients, to elucidate the association between SII and the rate of asymptomatic organ damage in HTN patients. Given the scarcity of relevant studies, a meta-analysis was not undertaken. Their study divided SII into quartiles, and the results showed that both the OR value and 95% CI increased as the SII value rose, with all OR values greater than 1. This proved that SII values were significantly positively associated with asymptomatic organ damage in HTN patients, serving as an independent predictor of such damage.

Uzun F *et al* (31) published a case-control study in 2022 (including 540 patients) that assessed the association between SII and MACE in HTN patients. Given the scarcity of relevant studies, a meta-analysis was not executed. In their study, the median and highest values of SII in the MACE group were significantly greater than those in the non-MACE group. This revealed that elevated SII was associated with an increased risk of MACE, serving as a potential associated factor for MACE.

Karakayali M et al. (22) published a case-control study in 2023 (including 272 patients) that appraised the association between SII and three types of HTN: dipper HTN, non-dipper HTN, and reverse dipper HTN. Patients with reverse dipper HTN had markedly higher mean SII values and experienced notably more physical damage compared to the other two groups, indicating an association between SII and HTN types. This result suggested that SII was a potential associated factor for the classification of HTN types.

## 4 Discussion

HTN affects a large portion of the global population and is associated with the incidence and mortality of CVDs. Therefore, it remains a critical public health issue (32–34). Even though traditional risk factors for HTN have been established, emerging evidence highlights the critical role of inflammation in the development and progression of this disease (6). Understanding the dynamic interplay between inflammation and HTN is crucial for identifying novel biomarkers that can enhance risk stratification and provide insights for targeted therapeutic interventions.

This meta-analysis systematically investigated the prognostic significance of SII in populations at risk for HTN as well as in patients diagnosed with HTN. In total, 17 studies were included, encompassing 187,195 patients. The results reveal that elevated SII levels could act as a potential associated factor for the diagnosis of HTN, and lead to an increased risk of MACE, all-cause mortality, CIMT, and asymptomatic organ damage in HTN patients. The publication bias assessment and sensitivity analyses have confirmed that our findings are reliable.

In order to assess the stability of the results and identify potential sources of heterogeneity, subgroup analyses were conducted by study design, sample size, geographic region, and age. The results indicate that SII exhibits favorable predictive performance across all subgroups; the I^2^ value in the American subgroup declined to 40%. This suggests that the heterogeneity observed in this study may primarily be related to regional differences.

For the association between SII and prognosis in patients with HTN, a meta-analysis was not conducted due to the scarcity of related studies. However, we find that compared to patients with low SII, those with high SII appear to have increased risks of mortality, CIMT, asymptomatic organ damage, and MACE. Therefore, a higher SII value is a potential associated factor for adverse outcomes of HTN. In the study by Karakayali M et al. (22), patients with reverse dipper HTN have markedly higher mean SII values and experience notably more physical damage compared to those with dipper HTN or non-dipper HTN. SII levels are negatively associated with hypertension types, indicating that SII is an independent predictor of the classification of HTN types.

Our study indicates a significant association between SII levels and the risk of HTN, and a close correlation is observed between SII levels and prognosis in HTN patients. This suggests that SII may play a complex role in the pathogenesis of HTN. Subgroup analyses were also performed to examine differences in the risk of HTN across various subgroups. The subgroup analysis by cutoff values of SII revealed that individuals with SII exceeding 500 × 10^9^l require heightened vigilance and active intervention, while conservative monitoring or treatment is needed for patients with low but persistently rising SII during follow up.

Some studies have identified a significant association between SII and potential complications of HTN. Xu et al. (35) have conducted a prospective cohort study utilizing data from the Dongfeng-Tongji cohort, including 13,929 patients. They find that SII is associated with an increased risk of stroke and other subtypes of stroke. They propose that SII could be a significant marker to clarify how thrombocytosis, inflammation, and immunity interact in the onset of CVDs in older and middle-aged populations. According to Zhao et al. (36), elevated SII levels are positively associated with an increased risk of ischemic stroke. These studies imply a significant association between SII and an elevated risk of ischemic stroke. Regular monitoring of SII may facilitate the early identification of individuals at high risk for ischemic stroke. Moreover, it may also help provide new evidence for primary prevention of ischemic stroke. SII not only serve as a potential factor in the development of HTN, but also is potentially associated with secondary complications of HTN, such as stroke, myocardial infarction, and coronary heart disease. As a result, SII is closely associated with both the incidence of HTN and the prognosis of HTN patients.

HTN is a cardiovascular syndrome characterized primarily by elevated systemic arterial blood pressure, mainly involving mechanisms like microvascular remodeling, aortic stiffening (3), autonomic nervous system imbalance, and RAAS (6, 37). Excess aldosterone modulates many components of the immune system, drives inflammation, and contributes to vascular, cardiac and renal damage, leading to aggravation of end-organ injury in cardiovascular and metabolic diseases. Primary HTN is associated with chronic low-grade inflammation and immune responses, which promote HTN by triggering vascular inflammation and microvascular remodeling (6). For instance, in the study published by Elisabetta Caiazzo et al. in 2022, the concentration of IL-6 is significantly associated with the risk of HTN. However, the specific mechanisms underlying this association remain incompletely elucidated. As primary responders of the immune system, NEUTs modulate vascular tone by expressing myeloperoxidase, a process closely tied to a decrease in the availability of nitric oxide (38). Additionally, elastase stored within NEUTs can facilitate the maturation of pro-inflammatory cytokines. Research has demonstrated a noticeable positive association between elastase levels and aortic pulse wave velocity, suggesting that elastase may be involved in arteriosclerosis (3, 39). At the same time, in a study published by Jing Wu et al., T cells, especially the cytokine IL-17A derived from T cells, contribute to atherosclerosis. Finally, NEUTs are capable of releasing neutrophil extracellular traps (NETs). This represents a novel mechanism of HTN identified in recent years. NETs, through the presentation of tissue factors (TF), induce thrombin production and platelet activation. Moreover, the NETs-TF-thrombin axis can exert a pro-fibrotic effect on endothelial cells (40). The aforementioned mechanisms together contribute to endothelial dysfunction and damage, thereby promoting the progression of HTN. LYMs are crucial in adaptive immunity and regulate blood pressure through the balance between inflammatory cytokines and regulatory T cells (Treg). Activated T LYMs can produce interleukin (IL) 17A and interferon-*γ*, inducing oxidative stress damage, and thereby leading to endothelial dysfunction (41). Treg exerts a protective effect in HTN and the damage to its target organs. This may be due to the secretion of IL-10, which limits angiotensin II-mediated oxidative stress and enhances vascular function (42). Primary HTN is a pro-thrombotic state characterized by elevated thrombin generation, potentially causing thrombotic complications, while PLTs are involved in this process (43). PLTs can facilitate NET formation through cell-cell contact or soluble mediators. NETs can also act as a scaffold for the adhesion, activation, and aggregation of PLT. The two processes mutually enhance each other, worsening the hypercoagulable state and promoting endothelial fibrosis (40, 44).

Studies have found that the possible mechanisms linking SII with the prognosis of HTN patients may be related to NEUTs, PLTs, and LYMs. NEUTs can release inflammatory mediators, resulting in endothelial dysfunction and vascular wall degeneration (45, 46). NEUT-derived proteases and reactive oxygen species lead to plaque instability and can increase thrombosis in the microcirculation (47, 48). Moreover, NEUTs have been proven to amplify tissue damage and inflammation in advanced atherosclerosis by triggering the lysis and death of smooth muscle cells (49). Furthermore, PLTs might release chemokines, pro-inflammatory cytokines, and PLT-derived growth factors, promoting the exhaustion of vascular endothelial cells (45). Moreover, NEUTs accelerate atherosclerosis and thrombosis through the proteolysis of PLTs and coagulation factor proteins, the release of prothrombin molecules, and monocyte infiltration, ultimately leading to an increased risk of cardiovascular events (45, 46). Conversely, LYMs can influence inflammatory cells and have a protective effect against atherosclerosis (45). Additionally, SII is associated with the severity of coronary stenosis, atrial fibrillation after coronary artery disease, contrast-induced acute kidney injury after coronary angiography (49–52), and adverse outcomes of ischemic stroke (53). The above studies suggest that elevated SII is a potential associated factor for poor prognosis of HTN.

Despite the substantial amount of data in this meta-analysis, there are several limitations that should be taken into account. Firstly, all eligible studies are conducted in Asia and the Americas, particularly in China, Japan, and the United States. Thus, our conclusions should be explained in this geographical background, and caution is needed for extending our findings to patients in Europe, Africa, and other regions. Further studies are essential to confirm the predictive performance of high SII in HTN in regions beyond Asia and the Americas. Secondly, most of the included studies adopt a retrospective design rather than a prospective design. The retrospective design might introduce potential confounders that may influence the reliability of the results. Additionally, the cut-off values of SII used in the studies are inconsistent. These cut-off values range from 267.4 to 869.5. The inconsistencies in the data may lead to inherent heterogeneity in our meta-analysis. To improve the reliability and comparability of future studies, a standardized cut-off value for SII should be established.

Furthermore, significant heterogeneity was observed in this meta-analysis (I^2^ = 100% for the continuous variable of SII and I^2^ = 88% for the categorical variable of SII). While subgroup analyses provide some insights into the source of heterogeneity [e.g., heterogeneity in the American subgroup decreased (I^2^ = 40%)], further investigation of the underlying sources of heterogeneity is necessary. High heterogeneity may stem from two main factors, biological and methodological, as well as from the interaction of multiple factors.

Several biological and demographic factors may contribute to heterogeneity. The included populations differed in baseline risk of hypertension (e.g., obesity, diabetes, chronic kidney disease, smoking status, and prevalence). These comorbidities independently influence systemic inflammation levels and SII values, thereby modifying the strength of the association between SII and hypertension. Ethnicity and genetic factors also significantly contribute to heterogeneity. The included studies were primarily based on Asian (Chinese, Japanese) and American populations, and differences in genetic susceptibility, lifestyle, and dietary patterns across ethnic groups significantly influence inflammatory responses and the risk of hypertension. Furthermore, heterogeneity may also be influenced by different hypertension phenotypes. The included studies may involve heterogeneous hypertension phenotypes (e.g., primary vs. secondary, well-controlled vs. refractory, and varying disease duration). Inflammatory burden and its relationship with SII may differ across subtypes. Our qualitative analysis suggests that SII levels differ between dipper HTN and non-dipper HTN. Finally, age and sex distributions also contribute to heterogeneity. Given the varying mean age across studies (Supplementary Table S1), sex ratios may also vary. Age and sex are known factors influencing immune function and inflammatory responses, potentially modifying the association between SII and hypertension.

Some methodological factors may also impact heterogeneity. First, cutoff values of SII vary across studies, which is a major source of heterogeneity. The definition of the cut-off value for high SII varies significantly across studies (267.4–869.5, Supplementary Table S1). This lack of a standardized cut-off value of SII directly impacts the categorization of exposure groups and the magnitude of the observed effect sizes (OR/SMD). The methods for selecting cutoff values are also inconsistent (e.g., median, quartiles, optimal ROC values). The significant heterogeneity observed, therefore, indicates that the relationship between SII and hypertension across diverse populations and methodological settings is complex. Although subgroup analyses suggest that regional differences may play a role, the primary driver is the interaction between biological factors (ethnicity, comorbidities, and hypertension phenotype) and inconsistencies in methodology (particularly the cut-off value of SII and study design). Due to this heterogeneity, it is infeasible to define universal clinical thresholds of SII. Future large-scale, prospective, multiethnic studies using standardized cut-off values of SII (perhaps based on large reference populations) and rigorously adjusting for confounding factors are urgently needed to clarify the precise role of SII in risk stratification and prognostic assessment of HTN.

The included studies exhibit contradictory findings: some studies affirm the predictive value of SII, while others refute it. For SII as a categorical variable, the results from Aljuraiban 2024 groups a and b, Ma L L 2024 group a, Sha S group, and ShiYia group crossed the 95% CI, yielding negative results, inconsistent with other studies. For SII as a continuous variable, the 95% CI did not cross zero in any case. Furthermore, as our analysis predominantly consists of retrospective studies, further research is warranted for validation.

Furthermore, due to a limited number of studies reporting critical prognostic outcomes (mortality, major adverse cardiovascular events MACE, carotid intima-media thickness CIMT, organ damage), the reliability of results was compromised. Future prospective studies specifically addressing these endpoints are required.

## 5 Conclusion

In conclusion, current evidence suggests that high SII levels may serve as a potential independent associated factor for diagnosing HTN in high-risk populations, and are strongly associated with adverse outcomes in HTN patients, including MACE, all-cause mortality, CIMT, and asymptomatic organ damage. Given the limited number of included studies and the retrospective design, more multi-center, large-sample prospective trials are required to validate the findings of this study.

## Data Availability

The original contributions presented in the study are included in the article/Supplementary Material, further inquiries can be directed to the corresponding author.

## Supplementary material

Supplementary Table 1 Basic characteristics of the included studies. The table presents the basic characteristics of the included studies, including study design, geographical location, population, sample size, gender, age, study design, BMI, SBP, DBP, SII cut-off, control and non-control groups, and various outcomes such as MACE and CIMT.
